# Identifying and Prioritizing Strategies for Developing Medical Tourism in the Social Security Organization of Iran: A SWOT-AHP Hybrid Approach

**DOI:** 10.18502/ijph.v49i10.4700

**Published:** 2020-10

**Authors:** Mirhojjat NAJAFINASAB, Lotfali AGHELI, Hossein SADEGHI, Sajjad FARAJI DIZAJI

**Affiliations:** Department of Economic Development and Planning, Faculty of Management and Economics, Tarbiat Modares University, Tehran, Iran

**Keywords:** Medical tourism, Social security organization (SSO), Hybrid analysis, Strategies

## Abstract

**Background:**

The present study aimed to identify and prioritize strategies for medical tourism improvement in the Social Security Organization (SSO) of Iran.

**Methods:**

Using Delphi method in the first step, we identify strengths, weaknesses, opportunities and threats that Social Security Organization of Iran faces in medical tourism during 2017–2018. In the second step we interviewed 100 medical and tourism experts to identify proper strategies using SWOT method and finally priori-tize strategies through the Analytic Hierarchy Process (AHP) by using Expert Choice software.

**Results:**

The application of SWOT technique indicated that the dominant strategy for the Iranian SSO should be conservative. It should overcome its internal weaknesses and exploit its external opportunities. The Iranian Social Security Organization should arrange appropriate marketing in target countries, invest in international medical/hospital standards, boost the communicative competence of personnel in SSO hospitals and create multilingual web-based information to introduce the capabilities and costs of the hospitals.

**Conclusion:**

Since the SSO’s weaknesses are more prominent than its strengths, thus SSO should plan to reduce its weaknesses to boost medical tourism. For this purpose, SSO should design coherent plans for attract medical tourists to the organization’s hospitals, attempt to upgrade hospital and medical centers standards, train the medical staff regarding linguistic and communicative skills, and run a user-friendly website to introduce its medical capabilities.

## Introduction

Health tourism is a term for organized trip that concentrates on medical treatments and the use of healthcare services. It includes medical tourism, wellness tourism and curative tourism (1). Treatment and recovery are main purposes of health tourism. Besides receiving medical services, leisure time activities are also included in the patient’s health package.

Treatment and recovery are main purposes of health tourism. Besides receiving medical services, leisure time activities are also included in the patient’s health package. The economic status and life difficulties can impose high level of stress on individuals. Health tourism is a choice for those who like to escape from daily stressful life. Improving health tourism can be considered as a national strategy for optimal use of domestic resources to increase the national income (2).

There has been a growing competition among different countries and particularly developing Asian countries to attract health tourists (3). The ease of seeking medical treatment and services overseas contributes to the globalization of the healthcare market (4).

According to the calculations, the foreign currency inflow by a health tourist is three times as much as a typical tourist (5). Considering the health tourism as a low-cost and high-yield industry, different countries are interested in developing tourism with an emphasis on health sector (6). Concerning its advantages in the field of health tourism including low costs, high-quality services, competent physicians, and having abundant natural attractions, Iran aims to make use of this opportunity (7).

Public infrastructure development strategy, human resources development strategy, information system and marketing development strategy, and product development strategy have been identified as the most important factors influencing the development of Iran’s medical tourism industry (8,9). Numerous studies have been conducted to recognize effective factors on medical tourism industry in world. A package of effective strategies has been proposed to develop medical tourism in Turkey. This package includes increasing competitiveness in medical services’ prices, easing the use of transportation and technological capabilities, promotion and marketing activities, increase in patients’ satisfaction during the initial welcoming, treatments and hospitality, touristic activities and departures, and employment of qualified, well-trained and fluent in foreign language staff in host hospitals (10). A SWOT analysis of medical tourism sector in India showed that as per international patients’ perspective, alternative treatment facilities like yoga have emerged out to be the biggest strength and cost of medical procedures which is comparatively lesser than developed countries are ranked as the second biggest strength (11).

The Social Security Organization (SSO) of Iran is the second largest provider of health services after the Ministry of Health and Medical Education. It covers more than 40 million of the population. This organization comprises 70 self-owned hospitals, 109 specialized specialist clinics, 93 general out-patient clinics, 83 specialized poly-clinics and 6 Day clinics across the country. In recent years, SSO has suffered from imbalance in revenue and expense accounts (12). Encouraging medical tourism is one of the revenue-generating approaches for SSO, since it has various capabilities in the field of hospital and clinical services including skilled physicians, well-equipped and up-to-date medical centers. In addition, if SSO deals with medical tourism, the inflows of foreign currency will increase. This study aimed to examine the pre-requisites, necessities, strengths/weaknesses, opportunities/threats facing the organization in dealing with medical tourism. This research aimed to identify and prioritize strategies of medical tourism development in SSO of Iran using SWOT-AHP hybrid method. Particularly, this research will answer the following questions:

- What are the internal/external factors affecting the development of medical tourism in Iranian Social Security Organization?
- What are the strategies for the development of medical tourism in Iranian Social Security Organization?
- What are the priorities of strategies for the development of medical tourism in Iranian Social Security Organization?

## Materials and Methods

This research uses a combination of SWOT and AHP methods. In the following, these methods are introduced briefly.

### SWOT Method

SWOT(Strengths- Weaknesses- Opportunities- Threats) technique is a tool for strategic planning used in many organizations to examine whether the goals are defined clearly or not, and whether all the relevant factors- positive or negative- are identified (13).

The comparison of strengths, weaknesses, opportunities and threats offers the final matrix including appropriate strategies applicable to the current status of the organization. External opportunities/threats are the existing events and processes outside the organization (14).

### AHP (Analytic Hierarchy Process) Technique

Thomas L. Saaty first introduced analytical Hierarchy Process in 1980 (15). This technique merges the experts’ opinions and evaluations and then transforms the complex decision making into a simple hierarchical system. It makes use of the scale-based evaluations for examining relative importance of pairwise comparisons among criteria (*16*).

### SWOT-AHP Hybrid Method

This method combines SWOT and AHP methods and constructs a matrix of strengths/weaknesses and opportunities/threats. This analysis is carried out in four steps as follows:

Step 1: The internal factors (strengths and weaknesses) and external factors (opportunities/threats) affecting development of medical tourism are identified using experts’ opinions (Delphi Group), and SWOT matrix is formed.
Step 2: The scoring and ranking of internal/external factors are carried out using experts’ opinions.
Step 3: To formulate and design the effective and appropriate strategy, *SWOT* matrix should be formed and following general strategies should be proposed:
  - *SO Strategy*: Making use of strengths to enjoy external opportunities
  - *WO Strategy*: Reducing internal weaknesses or improving ignored strengths to enjoy opportunities
  - *ST Strategy*: Making use of internal strengths to reduce external threats
  - *WT Strategy*: Reducing internal weaknesses to prevent external threats
Step 4: The strategies are prioritized using the *AHP* pairwise comparisons method.

Materials of this research are provided by designing and completing questionnaires. In this research we arranged interviews with 3 statistical societies. The first one includes 10 experts in tourism and medicine scope that we call it focus group. In the first phase, this population was responsible for identifying strengths/weaknesses and opportunities/threats using Delphi method. The second statistical population consists of medical care managers acting in the headquarters and other regional offices, SSO health experts, managers and experts of Tourism Holding of Social Security Organization.

The data was collected using 3 questionnaires: questionnaire No.1, distributed among members of the first statistical population (Focus Group), led to the identification of effective internal/external factors facing SSO about medical tourism. Questionnaire No.2 was designed according to the SWOT structure and was handed to 100 medical and tourism experts to define relative weights of weaknesses, strengths, opportunities and threats and to propose the appropriate strategies. Questionnaire No.3 was formed based on pairwise comparisons and Saaty scale (AHP) to prioritize the strategies defined in step 2 with the help of the decision-making group.

## Results

### Findings of the first phase

The findings of the first phase of the research are summarized in Table 1. It reports Delphi group opinions and classifies internal/external factors facing SSO in the field of medical tourism.

**Table 1:** Key strategic factors of Iranian SSO in the field of medical tourism

***Internal factors***	***External factors***
Strengths	Opportunities
S1: Low cost of surgeries in SSO medical centers compared to private centers	O1: Lower cost of surgeries in Iran compared to most developed countries, because of the higher exchange rates in Iran
S2: Paying more attention to invest in tourism industry by the organization	O2: Paying more attention to planning and investing in tourism industry by the government
S3: Existence of a large number of doctors with various expertise all over the country	O3: Rises in the number of European tourists entering the country because of the improvements in Iran’s foreign relations
S4: Numerous SSO medical centers all over the country	O4: Existence of religious cities and shrines in Iran as a complementary factor to medical tourism
S5: Existence of SSO residential centers in top tourist destinations of the country	O5: The legal obligation of the Ministry of Health and Medical Education for the development of medical tourism in the country
Weaknesses	Threats
W1: Lack of coherent and targeted programs for taking advantage of medical tourists’ presence in SSO	T1: Penetration of illegal mediators and mafia of power in medical tourism
W2: Low proficiency of service providers (doctors, nurses, etc.) in international languages	T2: Lack of effective laws on facilitating the admission of health tourists
W3: Lack of multilingual websites for introducing capacities and facilities of the organization’s medical centers and hospitals	T3: Extensive activities of private hospitals across the country to attract medical tourists
W4: Lack of valid international accreditation standards for hospitals such as JCI(Joint Commission International) and ACI (Accreditation Canadian International) in the organization’s hospitals	T4: Recent political tensions with Arab countries in the region
W5: Lack of marketing in the organization for attracting health tourists	T5: Emergence of new medical tourism destinations in the region such as Turkey, Jordan and UAE

Source: Research findings

### Findings of the second phase

In order to identify the factors which are out of the control of SSO, a list of opportunities and threats, and strengths/weaknesses of the organization is offered in Tables 2 and 3. We have provided this using questionnaire No. 2 by asking the opinions of medical care managers/experts, and other experts of tourism and medical sectors. External factors evaluation matrix (EFE) is a strategic tool used to evaluate firm existing strategies.

**Table 2:** External factors evaluation (EFE) matrix for the development of medical tourism in SSO

	***Factors***	***Weight***	***Score***	***Final Score***
Opportunities	O1: Lower cost of surgeries in Iran because of the higher exchange rates in the country	0.125	3.82	0.4775
	O2: Paying more attention to plan and invest in tourism industry by the government	0.075	2.6	0.195
	O3: Rises in the number of European tourists entering the country due to the improvements in Iran’s foreign relations	0.12	3.32	0.3984
	O4: Existence of religious cities and shrines in Iran as a complementary factor for attracting medical tourists	0.125	3.24	0.405
	O5: Legal obligation of the Ministry of Health and Medical Education for the development of medical tourism in the country	0.055	2.56	0.1408
Threats	T1: Penetration of illegal mediators and mafia of power in medical tourism	0.115	3.96	0.4554
	T2: Lack of exact and effective laws on facilitating the admission of health tourists	0.115	3.45	0.3968
	T3: Recent political tensions with Arab countries in the region	0.11	3.76	0.4136
	T4: Emergence of competitor medical tourism destinations such as Turkey, Jordan and UAE	0.105	3.39	0.3560
	T5: The competitive efforts of private hospitals in the country to attract medical tourists	0.055	2.94	0.1617
	Sum	∑=1	-	3.40

Source: Research findings

**Table 3:** Internal factors evaluation (IFE) matrix for the development of medical tourism in SSO

***S/W***	***Factors***	***Weight***	***Rank***	***Final score***
Strengths	S1: Low cost of health services in SSO medical centers compared to private centers	0.145	3.55	0.5148
	S2: Paying more attention to invest in tourism industry by the organization	0.075	2.51	0.1883
	S3: Existence of a large number of organization’s doctors with various expertise all over the country	0.10	3.09	0.309
	S4: Numerous SSO medical centers all over the country	0.115	3.05	0.3507
	S5: Existence of SSO residential centers (the organization’s hotels) in top tourist destinations of the country	0.065	2.75	0.1787
Weaknesses	W1: Lack of coherent and targeted programs for taking advantage of medical tourists’ presence in SSO	0.105	1.28	0.1344
	W2: Low proficiency of service providers (doctors, nurses, etc.) in international languages	0.095	2.12	0.2014
	W3: Lack of multilingual websites for introducing capacities and facilities of the organization’s medical centers and hospitals	0.10	1.98	0.198
	W4: Lack of valid international standards of hospital accreditation such as JCI and ACI in the organization’s hospitals	0.095	2.48	0.2356
	W5: Lack of marketing in the organization for attracting medical tourists	0.105	1.26	0.1323
	Sum	∑=1	-	2.42

Source: Research findings

In general, the purpose of forming EFE matrix is to evaluate whether the organization is capable of exploiting the opportunities and avoiding threats (17). In this matrix, first, the detected opportunities (O) and threats (T) are listed. Then a factor of importance or weight from 0 (not important) to 1 (very important) is assigned to each O/T. These weights indicate the relative importance of particular factors in the success of the organization under study. The sum of these factors must be 1. Then, each of them is scored from 1 to 4 (1 for a major threat, 2 for a minor threat, 3 for a minor opportunity and 4 for a major opportunity).

Table 2 exhibits the importance factor and rank of each opportunity/threat obtained through averaging experts’ opinions. In the next phase, the importance factor of each item is multiplied by its rank to obtain the final score. Likewise, the overall score of the organization is obtained. If this score is less than 2.5, the organization faces a threat. Otherwise, it means that the organization faces an opportunity.

According to Table 2 and EFE matrix, the final score of SSO in medical tourism is 3.40. This score indicates that the opportunities facing the organization are more than threats.

SSO can boost its capabilities by exploiting these opportunities. On the other hand, if appropriate measures are not taken for eliminating the threats, opportunities are more likely to be wasted in the long-term.

Besides analyzing the external factors, it is also necessary to examine the organization’s internal factors. Table 3 illustrates the importance factor/or weight and rank of each S/W obtained through averaging experts’ opinions. In the next phase, the overall score for the organization is obtained. If this score is less than 2.5, it means that the organization is weak in terms of the internal factors. Otherwise, the organization is strong in terms of these factors.

In Table 3, the final score resulting from IFE matrix is 2.42, which indicates that the strengths of the organization in the field of medical tourism are less than its weaknesses. Focus group approach has been used for providing a list of effective strategies in the field of medical tourism.

For this purpose, focus group session was held with 10 members of tourism managers and medical care experts. The members were offered with the SSO’s IFE and EFE matrices and they were asked for discussing the formulation of strategies. The output of this phase is a list of the organization’s strategies in the field of medical tourism. Table 4 displays four zones for aggressive, conservative, competitive and defensive strategies.

**Table 4:** SWOT matrix and SSO’s strategies in medical tourism

	***Strengths(S)***	***Weaknesses(W)***
	Aggressive strategies(SO)	Conservative strategies(WO)
Opportunities(O)	Introducing SSO hospitals as cheap destinations, especially for middle-class tourists (SO1) Planning for regional marketing in countries (especially Islamic countries) neighboring the provinces where are tourism destinations, (SO2) Designing hybrid travel packages of religious and medical tourism (SO3) (Strategy: Entering the medical tourism market of Iran)	Attempts towards targeted and coherent planning for attracting medical tourists to the organization’s hospitals (WO1) Attempts towards international standardization of medical centers concerning the qualification for accreditation certificates of ACI and JCI (WO2) Attempts towards training the medical staff regarding communications and international languages (WO3) Attempts towards running a comprehensive internet network for the organization’s medical centers/services (WO4) (Strategy: Using opportunities for reducing the organization’s weaknesses in medical tourism)
	Competitive strategies(ST)	Defensive strategies(WT)
Threats(T)	Active participation in the formulation of comprehensive laws in relevant organizations such as the Ministry of Health and Medical Education (ST1) Formulating inner-organizational regulations concerning medical tourism (ST2) Introducing price advantages in comparison to private hospitals (ST3) (Strategy: Using the organization’s strengths in medical tourism to reduce environmental threats)	Investment in promoting hoteling level in self-owned hospitals to compete with private hospitals (WT1) Creating an incentive system for the medical staff to enhance the quality of services provided for medical tourists (WT2) (Strategy: Conservative performance to prevent environmental threats)

Source: Research findings

### Findings of the third phase

Strengths (S), Weaknesses (W), Opportunities (O) and Threats (T) are linked to each other in 4 ways: SO, WO, ST and WT, which give the strategic options. Table 4 summarizes the SSO strategies in the field of medical tourism using the experts’ opinions about the importance factor of internal/external factors. The final internal/external factors (FIEF) matrix reveals which of the four zones of SWOT matrix will include aggressive, conservative, competitive and defensive strategies based on the SSO conditions (Table 5). The score is 2.42 for IFE and 3.40 for the EFE matrix, according to Tables 2 and 3, respectively. These scores indicate zone ӀӀ in Table 5.

**Table 5:** Final internal/external factors (FIEF) matrix of the Iranian SSO in medical tourism

	***IFE matrix final score***						
1	2.5			4		*EFE matrix final score*
II			I		4	
Conservative strategies			Aggressive strategies				
					
					
					2.5	
					
IV			III			
Defensive strategies			Competitive strategies		1	

Source: Research findings

This zone is characterized with weaknesses in terms of internal evaluation and opportunities in terms of external evaluation.

According to Table 5, Iran’s Social Security Organization should follow conservative strategies for boosting medical tourism. These strategies are as follows:

- Designing targeted and coherent planning for the attraction of medical tourists to the organization’s hospitals *(WO1)*
- Attempts towards international standardization of medical centers concerning the qualification for accreditation certificates of ACI and JCI *(WO2)*
- Training the medical staff regarding communications skills and international languages *(WO3)*
- Developing a comprehensive website for the organization’s medical centers *(WO4).*

### Ranking the strategies using AHP

This phase aimed to prioritize conservative strategies (WO). The criteria for priority setting are strengths(S)/weaknesses (W), opportunities (O)/threats (T) that have the highest weight in internal/external factors matrix.

The purpose, criteria and options are defined in *Expert Choice software,* and pairwise comparison is implemented in the matrix. According to four mentioned criteria, the available options to choose from are *WO1, WO2, WO3* and *WO4.*

In Table 6, pairwise comparison is carried out among the criteria, and their relative importance is evaluated.

**Table 6:** Matrix of relative importance of criteria

***Criteria***	***S***	***W***	***O***	***T***
S	1	3	2	3
W	1.3	1	4	5
O	1.2	1.4	1	2
T	1.3	1.5	1.2	1

Source: Research findings

**Fig. 1:**
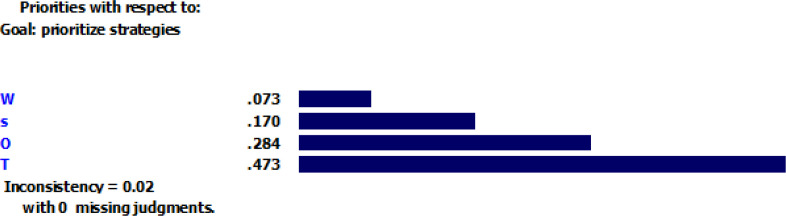
Inconsistency rate of the criteria Sources: Research findings

**Fig.2:**
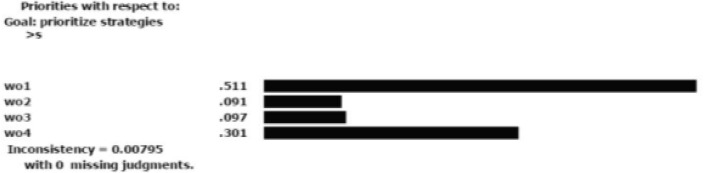
Impact of strengths’ weight on the options Sources: Research findings

**Fig.3:**
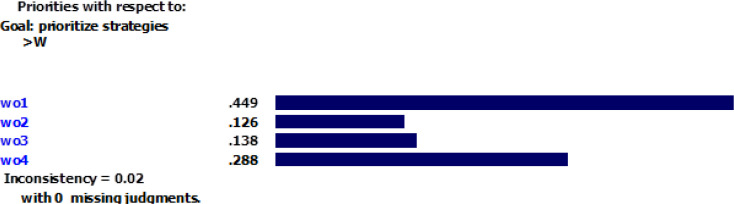
Impact of weaknesses’ weight on the options Sources: Research findings

**Fig. 4:**
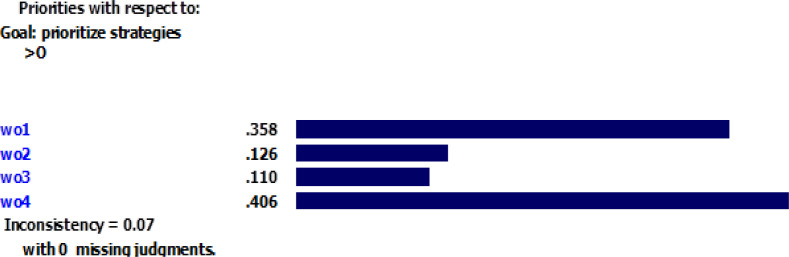
Impact of opportunities’ weight on the options Sources: Research findings

**Fig. 5:**
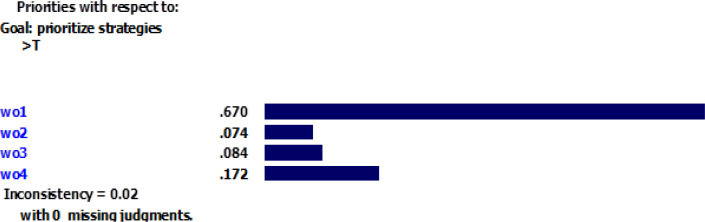
Impact of threats’ weight on the options Sources: Research findings

According to Fig. 1, the inconsistency rate for the criteria is 0.02, which indicates an acceptable precision for pairwise comparison. All strategies were compared in pairs considering each criterion. Figures 2–5 depicted the weight of each criterion and its impact on the options.

**Fig. 6:**
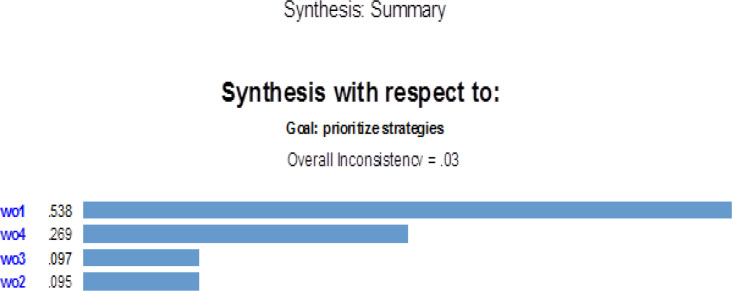
Options’ ranking and inconsistency rate of criteria Source: Research findings

According to the outputs of Expert Choice software reported in Fig. 6, the best option is *WO1* (Designing targeted and coherent planning for attracting medical tourists). Then comes *WO4* as the second priority, and the third priority is WO3, and finally WO2. Regarding the total inconsistency rate, which is equivalent to 0.03, pairwise comparisons are consistent, and research findings are valid.

## Discussion

This paper aimed to identify strategies for medical tourism development in SSO using SWOTAHP hybrid method in Iran. Regarding the external factors influencing medical tourism in SSO; the weight of the opportunities facing the organization is more than that of threats, which indicates potential opportunities for the organization to enjoy the advantages of medical tourism. The opportunities include low costs of surgery operations in Iran compared to other countries, the paying more attention to tourism industry by government, increase in the number of incoming tourists over recent years, existence of religious shrines/cities as factors attracting tourists and the legal obligation of the Ministry of Health and Medical Education for developing medical tourism. Our findings are compatible with similar work on medical tourism in India. Ajmera et al cite efficient infrastructure and hospital technology, skilled and expert workforce especially nurses in Indian hospitals, cheaper treatment in comparison to developed countries as medical tourism opportunities in India (11).

The scores of external and internal factors evaluation matrices (EFE and IFE) for SSO are 3.40 and 2.42, respectively. Therefore, the organization places in conservative zone (WO) in IE matrix based on SWOT analysis. As a result, four strategies of WO1, WO2, WO3 and WO4 are selected. By using AHP method and analyzing it in Expert Choice software, the WO1 strategy, i.e., attempts towards targeted and coherent planning for the attraction of medical tourists to SSO hospitals, is determined as superior to other ones. WO4, i.e., attempts towards running a comprehensive internet network for the organization’s medical centers/services) is ranked second. Finally WO2, i.e., attempts towards international standardization of medical centers concerning the qualification for accreditation certificates, and WO3, i.e., attempts towards educating the organization’s medical/tourism staff regarding communications and international languages, are simultaneously ranked third.

In general, from the view of technique used to analyze SWOT matrix at the field of medical tourism, this research is somehow comparable with previous studies. The proposed strategies are similar to medical tourism development strategies for Turkey (10). In particular, the employment of qualified, and bilingual or trilingual staff in host hospitals, and upgrading technological capabilities are regarded as common strategies in Iran and Turkey.

In a comparison of potentials of medical tourism in Iran and India, India relative to Iran is of more strengths and opportunities. Thus, Iran has been recommended to apply India’ experiences in expanding curative tourism (2). In this regard, health tourism development strategies include lowering prices of medical services, utilizing religious, cultural and linguistic similarities to attract health tourists from neighboring countries, advertising in the field of health tourism potentials and attractions. Upgrading quality and standard of providing medical services is a common strategy proposed by current study and as a practical solution to develop medical tourism, medical staff should be trained regarding communicative and linguistic skills. This point has not been considered in (2).

The results are also like findings of (9) focusing Tehran private hospitals, in which authors identify improving public infrastructure, developing human resources, upgrading information systems, promoting marketing and product development as main strategies for expanding medical tourism. Hence, this study recommends a “targeted and coherent plan” for attracting medical tourists to the SSO’s hospitals.

In Iran, most health/medical tourism researches have centered on national or local case studies, and no study has ever been done for the SSO’s hospitals. This research uniquely focuses on the selected hospitals of the SSO, and recommends targeted planning, accredited medical centers, trained and skilled medical staff, and on-line marketing at organizational level.

## Conclusion

In Iran’s development plans and its vision 2025, this organization has the capacity for running the industry. The results indicate the higher weight of the organizational weaknesses in medical tourism compared to its strengths in the field. The managers and decision-makers of the organization should focus their utmost attention on weaknesses of the organization. These include weak planning, lack of proficiency in international languages among the medical staff, lack of multilingual websites on medical services, lack of international standards of hospital accreditation and lack of marketing for attracting medical tourists.

In summary, four strategies are proposed for developing medical tourism by Social Security Organization as follows:

1. Designing targeted and coherent planning for attracting medical tourists to the organization’s hospitals (*WO1*)
2. Attempts towards international standardization of medical centers concerning the qualification for accreditation certificates of ACI (Accreditation of Canadian International) and JCI (Joint Commission International) (*WO2*)
3. Training the medical staff regarding communications and international languages (*WO3*)
4. Attempts towards running a comprehensive internet network for the organization’s medical centers (*WO4*).
